# Noninvasive assessment and quantification of tumour vascularisation using MRI and CT in a tumour model with modifiable angiogenesis – An animal experimental prospective cohort study

**DOI:** 10.1186/s41747-017-0014-5

**Published:** 2017-10-16

**Authors:** M. Mirus, S. V. Tokalov, G. Wolf, J. Heinold, V. Prochnow, N. Abolmaali

**Affiliations:** 1 Biological and Molecular Imaging, OncoRay – National Center for Radiation Research in Oncology, Dresden, Germany; 2 Department of Anaesthesiology and Intensive Care Medicine, University Hospital Carl Gustav Carus, University of Technology, Dresden, Germany; 3 Center for Thrombosis and Hemostasis, University Medical Center of the Johannes Gutenberg-University, Mainz, Germany; 4 Institute of Clinical Chemistry and Laboratory Medicine, Carl Gustav Carus Medical Faculty, University of Technology, Dresden, Germany; 5 Department of Neurology, Municipal Hospital Dresden-Neustadt, Dresden, Germany; 6 Clinic for Obstetrics and Gynecology, Klinikum Chemnitz, Chemnitz, Germany; 7 Department of Radiology, Municipal Hospital and Academic Hospital of the Technical University Dresden, Dresden-Friedrichstadt, Friedrichstrasse 41, 01067 Dresden, Germany

**Keywords:** MRI diffusion-weighted imaging, MRI time-of-flight, Computed tomography, Molecular imaging, Angiogenesis, Tumour microenvironment

## Abstract

**Background:**

To investigate vascular-related pathophysiological characteristics of two human lung cancers with modifiable vascularisation using MRI and CT.

**Methods:**

Tumour xenografts with modifiable vascularisation were established in 71 rats (approval by the Animal Care Committee was obtained) by subcutaneous transplantation of two human non-small-cell lung cancer (NSCLC) cells (A549, H1299) either alone or co-transplanted with vascular growth promoters. The vascularity of the tumours was assessed noninvasively by MRI diffusion-weighted-imaging (DWI), T2-weighted, and time-of-flight (TOF) sequences) as well as contrast-enhanced CT (CE-CT), using clinical scanners. As a reference standard, histological examinations (CD-31, fluorescent beads) were done after explantation.

**Results:**

Microvessel density (MVD) was higher in co-transplanted tumours (171 ± 19 number/mm^2^) than in non-co-transplanted tumours (111 ± 11 number/mm^2^; *p* = 0.002). Co-transplanted tumours showed higher growth rates and larger tumour vessels at TOF-MRI as well as larger necrotic areas at CE-CT. In co-transplanted tumours, DWI revealed higher cellularity (lower minimal ADC_diff_ 166 ± 15 versus 346 ± 27 mm^2^/s × 10^−6^; *p* < 0.001), highly necrotic areas (higher maximal ADC_diff_ 1695 ± 65 versus 1320 ± 59 mm^2^/s × 10^−6^; *p* < 0.001), and better-perfused tumour stroma (higher ADC_perf_ 723 ± 36 versus 636 ± 51 mm^2^/s × 10^−6^; *p* = 0.005). Significant correlations were found using qualitative and quantitative parameters: maximal ADC_perf_ and MVD (*r* = 0.326); maximal ADC_diff_ and relative necrotic volume on CE-CT (*r* = 0.551); minimal ADC_diff_ and MVD (*r* = −0.395).

**Conclusions:**

Pathophysiological differences related to vascular supply in two human lung cancer cell lines with modifiable vascularity are quantifiable with clinical imaging techniques. Imaging parameters of vascularisation correlated with the results of histology. DWI was able to characterise both the extent of necrosis and the level of perfusion.

## Key points

- Rat xenograft tumours with modifiable angiogenesis can be investigated using clinical scanners
- DWI characterises necrosis, perfusion, and vascularisation in tumours with variable vascularisation
- Differentiated views on ADC_diff_ give hints for histological traits of tumour vascularisation
- CE-CT and TOF-MRI visualise parameters reflecting vascularisation
- DWI, CE-CT, TOF-MRI noninvasively deliver data characterising specific parameters of tumour pathophysiology related to vascularity

## Background

Angiogenesis is of fundamental importance for growing tumours [1] and only by switching to an angiogenic phenotype, followed by the development of new vascularisation, solid tumours can overcome a critical size of 1–2 mm^3^ [2, 3]. Because of their own blood supply, cancer cells in solid tumours become independent from nutrition by diffusion of oxygen and nutrients from their surroundings. This lays the foundation for both the growth of the primary tumour and the spreading of tumour cells through the body. Characterisation of the vascularity of tissues in patients through imaging predicts response to therapy and correlates with prognosis [4].

Magnetic resonance imaging (MRI) of tumour vascularisation in vivo can be facilitated by time-of-flight (TOF) techniques for imaging the supplying tumour vessels and by diffusion-weighted imaging (DWI) [5] measuring the degree of the motion of free water in the imaging target [6–9] by calculating the apparent diffusion coefficient (ADC). Higher ADCs indicate fewer or less functional cell membranes frequently found in areas of necrosis or lower cellularity [10, 11]. Larger b-values in DWI acquisition generate a greater sensitivity for diffusion while with lower b-values gross motions within the target tissue, such as perfusion as well as extravascular and extracellular microcirculation, contribute increasingly to the received signal [11–13].

Contrast-enhanced computed tomography (CE-CT) delivers other parameters connected to vascularisation. Contrast enhancement is influenced by both vascularisation and the extravascular space and can be easily quantified [14]. However, results of dynamic CE-CT (DCE-CT) show considerable variations due to theoretical and technical limitations [15].

The reference standard for quantification of tumour vascularisation is immunohistology with different antibodies [16]. The use of intravenously administered fluorescent beads is another possibility to quantify vascularisation and perfusion [17]. Correlation with the reference standard is desirable to compare the results of different imaging modalities that investigate characteristics of vascularity.

The aim of this study was to investigate human lung cancer xenografts with different degrees of vascularity (tumour-induced and amplified), using different clinically available imaging techniques and to correlate these results with histology and immunohistology.

## Methods

To examine the influence of vascularisation on imaging parameters, a vascularisation-modifiable xenograft tumour model consisting of human cancer cell lines was used in this study. By the co-administration of vascular endothelial cells as vascular growth promoters, the vascularisation in tumours of the same cell line was manipulated [18–20]. This allows both to investigate the influence of vascular structures and vascular perfusion and to exclude influences of different cancer cell lines on imaging parameters. In this way, the differences related to the size of cells or nuclei, to the tumour stroma and microenvironment, to gene expression, and other cell-line characteristics should be minimised.

### Cells, animals, and tumour transplantation

The human non-small cell lung cancer (NSCLC) cell lines A549 and H1299 were investigated and rat glomerular endothelial (RGE) cells were used to modify vascularity in these tumours.

**Fig. 1:**
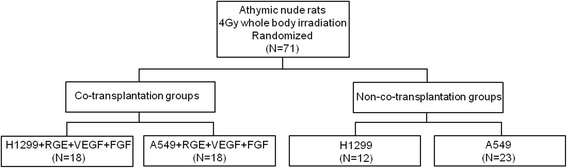
Stratification of four experimental groups with subcutaneously injected tumour cell suspensions and co-transplanted vascular growth promoters (tumour cell lines: H1299 and A549). RGE, rat glomerular endothelial cells; VEGF, vascular endothelial growth factor; FGF, fibroblast growth factor

The approval by the local Animal Care Committee was obtained in accordance with the institutional guidelines and the national animal welfare regulations. Four to 6-week-old athymic nude rats receiving food and water ad libitum were used. To enable tumour growth, all rats received one uniform whole-body irradiation of 4 Gy [21, 22]. Short anaesthesia during irradiation and CE-CT was performed by intraperitoneal injection of ketamine and xylazine [23], long anaesthesia during MRI was done with gas narcosis using desflurane. Tumour cells were transplanted 48 h after irradiation by subcutaneous injection of 200 μl of tumour cell suspension into the right lower limb at the femur. The rats in the animal experimental prospective cohort study were assigned randomly to one of four groups (Fig. 1) and correspondingly received 5 × 10^6^ tumour cells, 2 × 10^6^ RGE cells, 160 ng recombinant human vascular endothelial growth factor 165 (rHu-VEGF-165), and 320 ng recombinant human fibroblast growth factor b (rHu-FGF-b). After injection, animals were inspected every 2 days and the tumour’s length and width were measured by a caliper. As soon as any tumour had reached a size of 20 mm in one dimension, this tumour-bearing rat was subjected to the multimodal imaging protocol.

### Imaging techniques

The imaging of tumours included MRI, DWI, CE-CT, and histological examinations. For CE-CT a positron emission tomography (PET)/CT Biograph 16 (Siemens AG, Erlangen, Germany) was used. A catheter implanted into the jugular vein was used for intravenous application of contrast material (Ultravist 370, Bayer Vital GmbH, Leverkusen, Germany) and beads. The CT protocol was: unenhanced CT scan; CE-CT after intravenous injection of 1 ml of contrast material using a small-animal injection pump (Medtron, Saarbrücken, Germany). The CT scan started 20 s after injection start. The scan specifications were 80 kV and 100 mAs; 512^2^ pixels with a voxel resolution of 420 μm × 420 μm × 750 μm.

For MRI, a 1.5-T system and an eight-channel clinical knee coil (Magnetom Avanto, Siemens AG, Erlangen) were used. Rats were placed on a custom-made bed for gas narcosis. The following sequences were performed: T2-weighted turbo spin-echo (voxel size 0.6 mm^3^, time of repetition [TR] 20.12 ms, time of echo [TE] 6.04 ms, acquisition time 10 min and 53 s); three-dimensional TOF (voxel size 0.3 mm^3^, TR 40 ms, TE 8.01 ms, acquisition time 21 min and 46 s); DWI (voxel size 1.8 mm^3^, TR 6300 ms, TE 94 ms, three orthogonal diffusion gradient directions, b-values 0, 50, 100, 150, 200, 250, 300, 500, 750, and 1000 s/mm^2^, acquisition time 17 min and 45 s).

### Histology and immunohistology

Two hundred microlitres of suspension containing 2.4 × 10^7^ fluorescent beads (diameter 2.5 μm, excitation wave length 633 nm; G. Kisker GbR, Steinfurt, Germany) were injected via the jugular vein. After imaging and after two min of beads’ circulation the rats were sacrificed and the organs as well as the tumours were removed [17].

Representative slices (slice thickness 10 μm) of the liver and kidneys (to verify appropriate application of beads) as well as the tumours were stained with hematoxylin-eosin. Tumour slices were additionally stained with CD-31 antibody to quantify microvessel density (MVD). The quantification of beads embolised into the vascularity of tumours was examined by fluorescent microscopy.

### Image analysis

Histology and bead counts (beads: number of beads/field of view) served as standard of reference and were analysed within the tumours, the liver, and the kidneys. For CD-31 staining, tumour slices were digitally recorded at 20-fold magnification and the number of CD-31 positively stained structures were evaluated as published before, with MVD measured as the number of stained structures/mm^2^ [16].

For analysis of CE-CT data, a contour was drawn around the contrast-enhanced tumour in all slices, including the outer rim of the tumour, the skin, and unenhanced lacunas but excluding bone and vessels without contact to the tumour. Limits of density values for analysis of the entire tumour were -50 ≤ HU ≤ +350. These contours were copied to the plain CT series and mean HU values in the tumours before and after contrast injection were measured.

The MRI protocol contained morphological and functional techniques. T2-weighted imaging served for co-localisation and tumour volumetry, the latter was obtained by manual segmentation. For visualisation of vessels, TOF-angiography was done and an ordinal scale was applied to the resulting three-dimensional image. The comparison to the three-dimensional datasets of the opposite femoral region was quantified according to the following ordinal scale: tumours received zero points if no differences in the visualisation of the vessels were detectable, one point if an increased vascularisation was visible, e.g. by separate short vessels, and two points if large circumscribable vessels travelled through the tumour. We did not use contrast-enhanced angiography studies (neither CT angiography nor MR angiography) for qualitative vessel visualisation since contrast bolus timing for appropriate imaging is dependent on circulation parameters that vary to a very high degree in sedated rats. To evaluate the quantity of small vessels and related motion in the extracellular space, the acquired b-values of DWI were utilised to separate apparent diffusion coefficients (ADCs) [11] for diffusion (ADC_diff_) and perfusion (ADC_perf_) [24]. Basically, for each slice, the images with b-values of 500, 750, and 1000 s/mm^2^ were aggregated to calculate eight-bit coloured ADC_diff_ maps and images with b-values of 50, 100, and 150 s/mm^2^ were aggregated and subtracted from ADC_diff_ maps to calculate eight-bit coloured ADC_perf_ maps. These were registered to the T2-weighted image stacks and the contours of the tumours were copied to the ADC maps to extract the minimum, maximum, and mean ADC_diff_ and ADC_perf_.

### Statistical analysis

All statistical analyses were carried out with PASW 18 (Predictive Analytics SoftWare, IBM, Armonk, NY, USA) by comparisons between co-transplanted and non-co-transplanted tumours separated for each cell line as well as averaged for both cell lines. The Kolmogorov-Smirnov test was used to test for normality. The Mann–Whitney *U* test was used to analyse non-normally distributed data, whereas the *t* test was used for normally distributed data. Spearman’s rank correlation coefficients were calculated to evaluate correlations. The χ^2^ test was used to compare the results of TOF sequences between the groups. A *p* value lower than 0.050 was considered to indicate a statistically significant difference.

## Results

### Cells, animals, and tumour transplantation

**Fig. 2:**
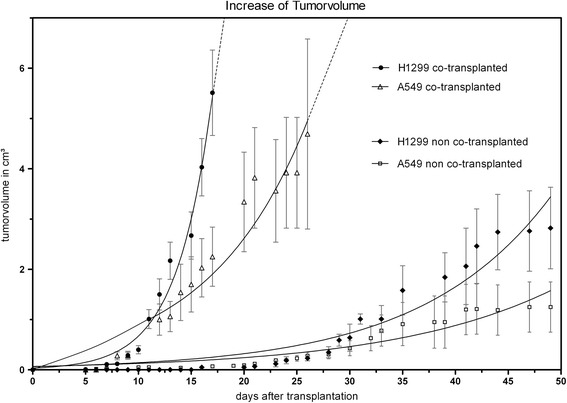
Growth of xenograft tumour volumes of the non-small-cell lung cancer (NSCLC) cell lines A549 and H1299. Tumours co-transplanted with rat glomerular endothelial (RGE) cells, recombinant human vascular endothelial growth factor 165 (rHu-VEGF-165) and 320 ng recombinant human fibroblast growth factor b (rHu-FGF-b) showed higher-volume growth as compared to non-co-transplanted tumours

**Fig. 3:**
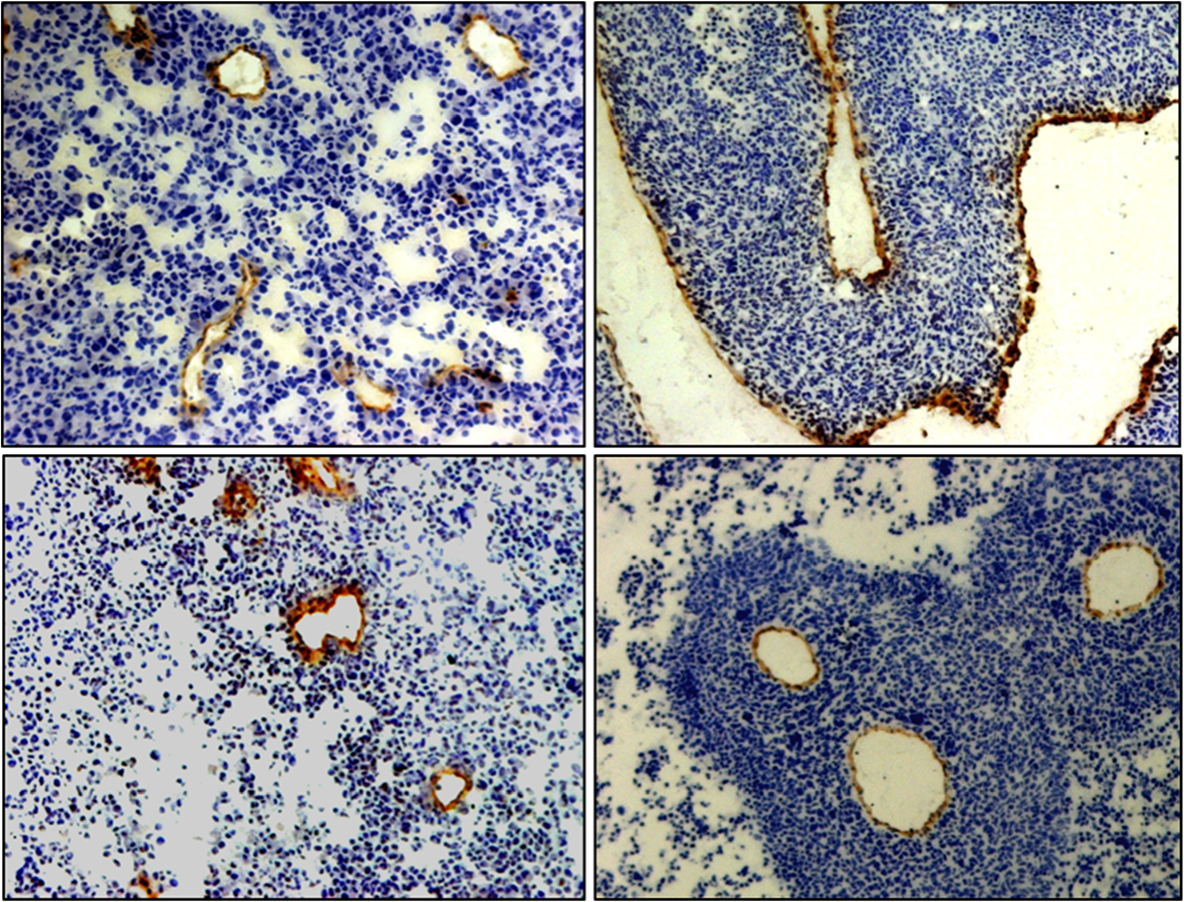
Vessel marker CD-31-positive structures (brown) in tumour sections. While the upper and lower rows depict images from cell lines A549 and H1299, respectively, the left and right columns depict images for non-co-transplanted and co-transplanted tumours, respectively. Right column: clusters of compact vital tumour cells enclose to vessels in both co-transplanted tumours. Left column: much looser cell clusters enclose to smaller CD-31-positive structures (brown) in both non-co-transplanted tumours

Both tumour cell lines H1299 and A549 revealed significantly faster growth of the tumours in the co-transplanted group as compared to the non-co-transplanted group (Fig. 2). Tumours in animals with co-transplantation reached imaging size earlier (Table 1). While bead embolisation in reference organs was not different between the groups, the vascularisation in tumours quantified by MVD and count of beads embolised into the tumours showed significant differences (Table 1). MVD and bead count were higher in co-transplanted tumours. For both cell lines, the central regions of the co-transplanted tumours often showed clusters of necrotic regions as well as vessels with larger diameters often closely connected to clouds of CD-31-positive small vessels (Fig. 3). Both necrotic areas and CD-31-positive vessels with a wide diameter were only rarely found in the centre of non-co-transplanted tumours (Fig. 3).

**Table 1:** Growth and histology

		H1299			A549			All tumours		
		Non-co-transplanted	co-transplanted	*p* value	Non-co-transplanted	co-transplanted	*p* value	Non-co-transplanted	co-transplanted	*p* value
In vivo growth duration	[days]	49.0 (20)	18.0 (11)	<0.001	45.0 (10)	23.5 (15)	<0.001	45.0 (7)	22.0 (12)	<0.001
In vitro beads	[number/FOV]	1.1 (0.28)	1.3 (0.17)	0.009	1.1 (0.22)	1.3 (0.23)	0.032	1.1 (0.25)	1.3 (0.22)	0.001
In vitro MVD	[number/mm^2^]	89.7 (65.49)	203.1 (185.49)	0.036	114.6 (81.98)	153.4 (112.75)	0.018	110.8 (63.30)	171.4 (120)	0.002

*FOV* field of vision*, MVD* microvessel density

Overview of *in vivo* growth velocity until the imaging size of 2 cm was reached and *in vitro* histology and immunohistology. First and second column show results separately for both cell lines, in the third column data for both tumour cell lines were merged. Median (interquartile range)

### CT imaging

**Fig. 4:**
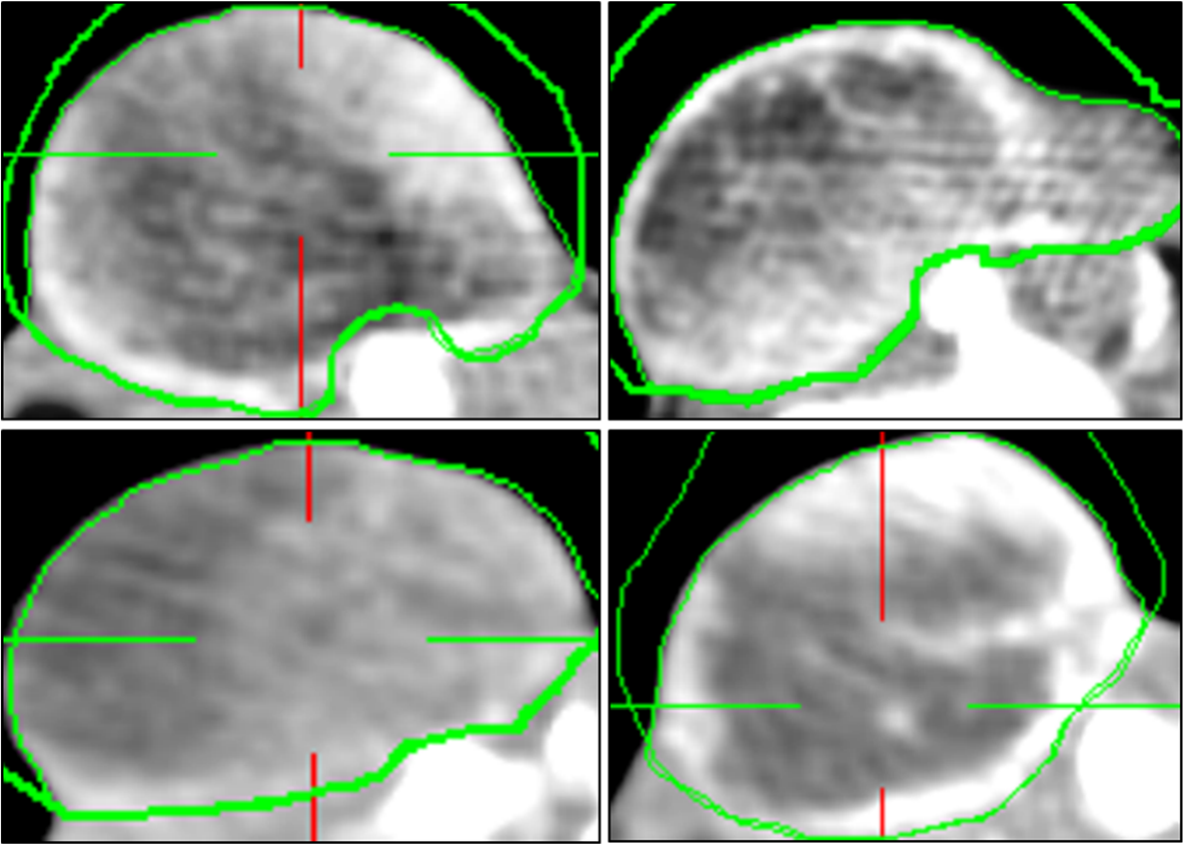
Details of xenograft tumours and relevant regions of interests as acquired by contrast-enhanced computed tomography (CE-CT). While the upper and lower rows depict images from cell lines A549 and H1299, respectively, the left and right columns depict images for non-co-transplanted versus co-transplanted tumours, respectively. Left column: CT images of tumours from the non-co-transplantation groups show comparatively homogenous hypodense and adjacent solid areas with limited contrast enhancement after intravenously administered contrast injection. Right column: CT images of tumours from the co-transplantation groups show central areas predominantly liquid/cystic and strong, most likely vascular, enhancement radiating into the tumour mass

On unenhanced CT scans, co-transplanted tumours revealed a trend towards lower HU values (30.5 ± 0.5 HU, mean ± standard deviation), while non-co-transplanted had higher HU values (32 ± 1 HU). After intravenously administered contrast injection (Fig. 4), co-transplanted tumours showed a higher contrast uptake (120 ± 2.2 HU) than non-co-transplanted tumours (111 ± 2 HU). This difference was highly significant (*p* = 0.001).

### MRI

#### T2-weighted and TOF sequences

Higher volumes in the co-transplanted tumours than in non-co-transplanted tumours were found (Table 2). TOF sequences allowed the visualisation of vessels in all xenograft tumours and revealed larger vessels in co-transplanted tumours (Table 2).

**Table 2:** Study results magnetic resonance imaging (MRI) data

		H1299			A549			All tumours		
		Non-co-transplanted	Co-transplanted	*p* value	Non-co-transplanted	Co-transplanted	*p* value	Non-co-transplanted	Co-transplanted	*p* value
Tumour volume T2	[cm^3^]	1.6 (2.4)	3.8 (4.8)	0.001	0.2 (0.3)	4.4 (6.5)	0.000	0.2 (1.7)	4.0 (5.2)	<0.001
TOF	Numeric scale	1.3 ± 0.1	1.7 ± 0.1	0.009	1.2 ± 0.1	1.4 ± 0.1	0.009	1.2 ± 0.1	1.5 ± 0.1	0.006
Maximal ADC_diff_	[mm^2^/s × 10^−6^]	1473 (616)	1672 (597)	0.047	1206 (396)	1714 (674)	0.005	1320 (441)	1695 (546)	<0.001
Minimal ADC_diff_	[mm^2^/s × 10^−6^]	311 (385)	149 (77)	<0.001	348 (199)	173 (135)	<0.001	346 (224)	166 (97)	<0.001
Maximal ADC_perf_	[mm^2^/s × 10^−6^]	1712 (830)	2994 (305)	<0.001	1612 (614)	2999 (657)	<0.001	1616 (661)	2999 (476)	<0.001
Mean ADC_perf_	[mm^2^/s × 10^−6^]	568 (149)	722 (106)	0.001	646 (262)	723 (184)	0.203	636 (208)	723 (144)	0.005

*In vivo* MRI quantitative results of T2 volumetry, time-of-flight (TOF) and apparent diffusion coefficient (ADC). First and second column show results separated for both cell lines, third column tumour cell lines merged together. Median (interquartile range); TOF: mean ± SD

#### DWI, ADC_diff_, and ADC_perf_ values

**Fig. 5:**
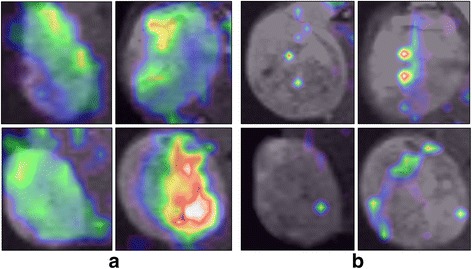
**a.** Details of xenograft tumours: T2-weighted images fused on corresponding colour-coded apparent diffusion coefficient (ADC) maps. Upper row: tumours from cell line A549; lower row: tumours from cell line H1299. Left column: non-co-transplanted tumours; right column: co-transplanted tumours. Left column: the image fusion of the ADC_diff_ maps of non-co-transplanted tumours reveals comparatively homogenous and intermediate values of diffusion (*gree*n). Right column: the image fusion of the ADC_diff_ maps of co-transplanted tumours reveals the existence of extreme values of diffusion (*red* = high; *dark blue* = low). Range of ADC_diff_ (mm^2^/s × 10^−6^) in the tumour were: top line left, 312–1593; top line right, 31–1586; lower line left, 55–1920; lower line right, 30–2584. **b.** Details of xenograft tumours: T2-weighted images fused on corresponding colour-coded ADC maps. Upper row: tumours from cell line A549; lower row: tumours from cell line H1299. Left column: non-co-transplanted tumours; right column: co-transplanted tumours. Left column: the image fusion of the ADC_perf_ maps of non-co-transplanted tumours reveals only very few vessels that are large enough to be visualised (*green dot*). Right column: the image fusion of the ADC_perf_ maps of co-transplanted tumours reveals larger perfused vessels (*green linear structures*). Range of ADC_perf_ (mm^2^/s × 10^−6^) in the tumour were: top line left, 3–3502; top line right, 1–3977; lower line left, 1–2733; lower line right, 2–3920

All ADC parameters were acquired in the kidneys in all groups for reference and no significant difference for any ADC parameter was found (data not shown). DWI revealed significantly lower minimal ADC_diff_ values and significantly higher maximal ADC_diff_ values in co-transplanted tumours as compared to non-co-transplanted tumours (Table 2, Fig. 5a). At DWI perfusion analysis, significantly higher mean and maximal ADC_perf_ values were found (Table 2, Fig. 5b) in co-transplanted as compared to non-co-transplanted tumours. The mean ADC_perf_ values in H1299 tumours were significantly larger in co-transplanted than in non-co-transplanted tumours (*p* = 0.001) while in A549 tumours this difference was not significant (Table 2).

### Correlation analyses

**Fig. 6:**
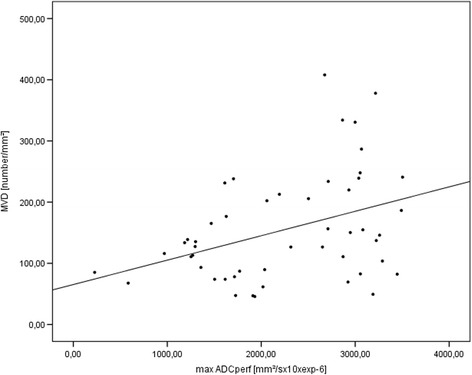
Spearman’s rank correlation between microvessel density (MVD) and maximal ADC_perf_; *p* = 0.010; *r* = 0.326

**Fig. 7:**
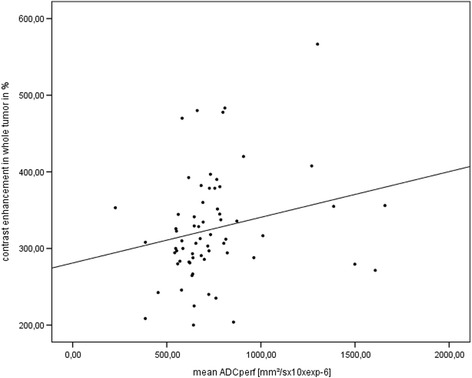
Spearman’s rank correlation between contrast enhancement in whole tumour in contrast-enhanced computed tomography and mean ADC_perf_; *p* = 0.009; *r* = 0.294

**Fig. 8:**
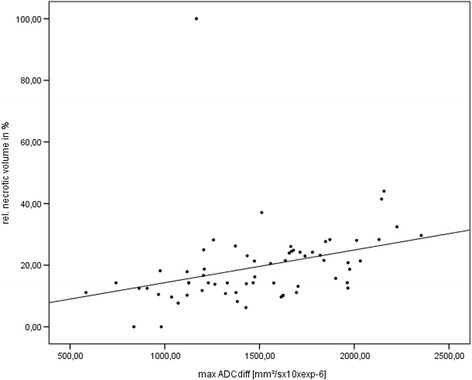
Spearman’s rank correlation between relative necrotic volume on contrast-enhanced computed tomography and maximal ADC_diff_; *p* < 0.001; *r* = 0.551

**Fig. 9:**
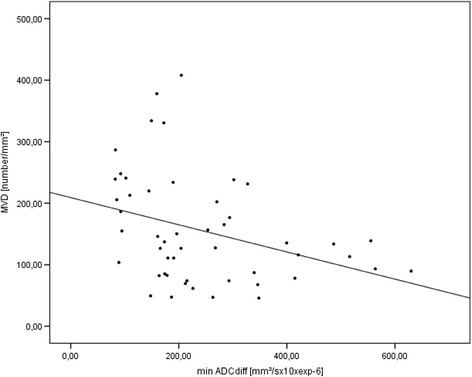
Spearman’s rank correlation between microvessel density (MVD) and minimal ADC_diff_; *p* = 0.002; *r* = −0.395

The following correlations were found to be significant considering all tumours: the correlation between maximal ADC_perf_ and MVD (*p* = 0.010; *r* = 0.326; Fig. 6), the correlation between mean ADC_perf_ and contrast enhancement in whole tumour in CE-CT (*p* = 0.009; *r* = 0.294; Fig. 7), the correlation between maximal ADC_diff_ and relative necrotic volume estimated in CE-CT (*p* < 0.001; *r* = 0.551; Fig. 8) and the correlation between minimal ADC_diff_ and MVD (*p* = 0.002; *r* = −0.395; Fig. 9). For technical reasons, the computer-based calculation of the necrotic volume at CE-CT resulted in a value of 100% for one rat which is the outlying value in Fig. 8. Excluding this data point from the correlation analysis increases the correlation between the maximal ADC_diff_ and relative necrotic volume estimated in CE-CT to *p* < 0.001; *r* = 0.594 (Spearman’s rank).

## Discussion

The aim of this study was to evaluate imaging parameters quantifying vascularity in human cancer xenografts with two levels of vascularisation. The amount of vascularisation was determined ex vivo by a reference standard, i.e. specific histological and immunohistological methods. To provide translational results, these tumours were investigated *in vivo* with scanners used in clinical patient care. Clinically applied imaging techniques were utilised and optimised for small-animal imaging.

The selected human lung cancer cell lines are frequently used in preclinical xenograft research, typically on mice. In this study the authors decided to use a rat model. These animals can carry larger tumour sizes that mandate induction of relevant vascularisation to overcome restrictions of oxygen diffusion distance and additionally will develop relevant amounts of necrosis. Moreover, these tumour sizes are appropriate for evaluation with clinical scanners and imaging protocols. Two different lung cancer cell lines were utilised and both of them were investigated either with or without co-injection of vascular growth promoters (i.e. vascular growth factors and rat endothelial cells) creating two different levels of vascular development in the growing tumours of both cell lines. Since tumour vascularity in xenograft tumours is developed by the host animal, rat vascular growth factors were used to modify vascularity. Better tumour vascularisation causes a better supply of oxygen and nutrients which results in a faster growth rate. Nevertheless, growth rates are also dependent on the pathophysiological properties of the individual tumour cell line. Accordingly, after tumour and vascularity-dependent growth times tumours were evaluated and different levels of vascularity were quantifiable with clinical scanners and techniques.

Co-transplantation resulted in faster tumour growth. Since the amount of transplanted tumour cells was constant, the faster growth rate can be explained by a better supply of oxygen and nutrients as well as an improved evacuation of lactate and end products of metabolism due to better vascularisation. An important condition for fast tumour growth is the angiogenic switch that is dependent on endothelial precursor cells [25]. Beside all other influences, co-transplantation with vascular growth factors and rat endothelial cells increased the probability of a successful switch to an angiogenic phenotype which led to better vascularisation in these tumours. This was proven in our study by the increases of MVD and the larger amount of embolised beads within the tumours. Interestingly, the co-transplanted tumours grew so fast that the development of central necrosis was higher in this group even though the number of larger vessels was also higher. In fact, CE-CT showed wide hypodense areas within a contrast-enhancing tumour parenchyma at the tumour rim and around larger central vessels in co-transplanted tumours. Using TOF sequences, co-transplanted tumours revealed the development of larger vessels in contrast to non-co-transplanted tumours. In summary, better vascularisation boosts fast growth which at the same time can increase necrosis by uncoupling regions from blood supply due to fast growth. Conversely, this means that regions of necrosis may be an indirect indicator of better vascularisation.

Adding a functional MRI technique, like quantitative DWI, with the calculation of ADC values, increases the diagnostic information. ADC_diff_ describes various properties of tumours. Higher ADC_diff_ values indicate higher diffusion; that is equating with fewer or less functional cell membranes frequently found in areas of necrosis or lower cellularity. As known from biology and neurology, necrosis is a continuum extending from viability to cell to tissue death. Therefore, a differentiated view on the ADC_diff_ is required since the latter allows for detecting different levels of necrosis.

Furthermore, ADC_diff_ conveys indirect information concerning the viable part of the tumours and the condition of the tumour microenvironment [26]. The peak value of ADC_diff_ (high maximal ADC_diff_) reflects the degree of necrosis, whereas the nadir of ADC_diff_ (low minimal ADC_diff_) informs about the condition and cellular density of the viable tumour cells. Both permit direct and indirect interpretations on the level of vascularisation, blood supply, and the rate of growth. The range of the ADC_diff_ values provides an additional source of information for a differentiated interpretation of ADC_diff_. The maximal ADC_diff_ in a tumour may reflect the extent of necrosis in certain regions. As mentioned before, better vascularisation induces faster tumour growth that may impede the balance between nutrient supply and nutrient demand which may trigger regional critical shortness in blood supply leading to increased necrosis [27, 28]. In addition, fast cellular growth rate is paralleled by the unorganised development of pathological vessels leading to critical shortage of oxygen in highly vascularised tumours. Such shortage leads to necrosis in relevant parts of larger tumours. The formation of regions with severe necrosis in better-vascularised tumours can be the consequence. Such necrosis is not seen in smaller tumours [29] because these tumours show a slower growth rate with more adequate vascular development and do not develop critically supplied areas [28]. The histological findings in this study supported this fact and are in agreement with the current knowledge on the pathophysiology of tumour growth. In highly vascularised, large tumours, wide-diameter vessels, which lack further small branches, were found traversing through necrotic regions. Thus, nutrition in these regions was limited to diffusion from one large single vessel. In this respect, better-vascularised tumours exhibited areas with more necrosis. These areas were formerly viable tumour regions that lost their oxygen supply and became necrotic. This situation was found less often in slower-growing tumours. To sum up, tumours with better vascularisation exhibit both severe necrosis and well-vascularised areas. Consequently, the existence of large necrotic areas in a tumour can be an indirect indicator for good vascularisation. The differentiated view on ADC_diff_ could detect this indicator. More precisely, the maximal ADC_diff_ (high maximal values in a tumour) suggests the existence of areas of high necrosis which is more often found in better-vascularised tumours. Hence, a higher maximal ADC_diff_ suggests better-vascularised tumours. The results of this study support this fact. DWI is capable of detecting these differences and was already found to correlate with MVD [30]. The ADC_diff_ is much higher in better-vascularised tumours because of the existence of highly necrotic areas exhibiting high diffusion values [31]. The peak value of ADC_diff_ (maximal ADC_diff_) indicates focal areas of severe necrosis found in tumours with faster growth due to better vascularisation. The latter was insufficient at some point of tumour growth resulting in critical shortage of oxygen and nutrients and consecutive development of necrosis. This may indicate a poorer prognosis as Marconi et al. recently showed in women with advanced-stage cervical cancer [32].

The interpretation of the minimal ADC_diff_ should also be meaningful. The two major factors for impeded diffusion as estimated by the lowest values of ADC_diff_ are the severely reduced intracellular space (due to more organelles and larger nuclei) and extracellular space (due to high proliferation rate) in viable tumour tissue. In general, the lower ADC_diff_ values correspond to higher cellularity and more compact tumour tissue. Impeded diffusion in tumours occurs if tumour cells can proliferate and form their typical tumour microenvironment. However, such proliferation requires oxygen and nutrient supply and, therefore, the existence of a vascular system within the tumour. As found herein, a low minimal ADC_diff_ may indirectly indicate well-vascularised areas. The available academic literature gathered contradictory results related to this topic. In women suffering from cervical cancer, Nakamura et al. found that low ADC values in the tumours were related to poor prognosis [33] which supports the findings in this study, since patients with highly vascularised tumours frequently exhibit a poorer prognosis.

In summary, the range of the ADC_diff_ is related to high vascularisation. More precisely, higher maximal ADC_diff_ values are associated with high necrosis as a consequence of very fast tumour growth while very low minimal ADC_diff_ values are associated with high cellularity, dense cell-cell contacts, and high protein expression as a consequence of good proliferation. Both fast growth and good proliferation may be surrogate parameters related to better vascularisation.

The ADC_perf_ is capable of detecting perfusion in tissues directly and is related to vascularisation in tumours. In this tumour model, highly vascularised and well-perfused tumours exhibited higher ADC_perf_ values, indicating increased tumour vascularisation. This was shown in both cell lines and was independent from the influence of the histological cell type. The results of this study show that the separation of the ADCs can provide physicians with more information. However, in addition to the suggestion by Koh et al. [12] that separating different b-values may contribute to this, it was shown herein that the differentiated analysis of minimal and maximal ADC values may increase the value of the imaging [34, 35].

DWI can be used in extension to histology and other imaging modalities. The evaluation of vascularisation in tumours can be improved if different methods are used in combination. ADC_diff_, ADC_perf_, MVD, and other methods detect correlated characteristics of vascularisation in tumours. The low, but significant, correlation between MVD and maximal ADC_perf_ supports the idea that both parameters are influenced by vascularisation properties. The reason for the comparatively weak correlation might be that both parameters are influenced by different aspects of vascularisation. MVD is predominantly influenced by the existence of vessels (which do not necessarily have to be well perfused), whereas the ADC_perf_ is mainly influenced by the level of perfusion (which is not necessarily linked to the existence of intact vessel walls).

The correlation with the minimal ADC_diff_ values is also in line with this concept. Well-vascularised tumours developed areas with high proliferation and high cellularity which may account for the correlation of MVD and the minimal ADC_diff_. This indicates that perfusion and vascularisation in tumours affect the DWI values obtained which was confirmed by the histological results of this study. Much more than the ADC_perf_, the minimal ADC_diff_ and the MVD are influenced by further aspects of vascularisation. The authors speculate that the minimal ADC_diff_ is linked indirectly to vascularisation, whereas MVD is connected much more closely to vascularisation. While this might explain the small magnitude of this correlation, a small, but significant, correlation can be also meaningful. As described before, highly vascularised fast-growing tumours reveal both well-perfused and necrotic areas. The correlation between CE-CT and DWI implies that both methods capture this situation. Necrotic areas were detected by high maximal ADC_diff_ values. CE-CT visualised larger, comparatively necrotic, volumes and stronger contrast enhancement in vascularised areas. In this case, both parameters – the maximal ADC_diff_ and the relative necrotic volume in CE-CT – are directly influenced by the same factor: the level of necrosis. Both diffusion and contrast enhancement increase with increasing levels of necrosis. Accordingly, a moderately significant correlation was found. Thus, DWI and CE-CT complement each other and extend the insights in the tumour biology concerning vascularisation.

One limitation of this study is the utilisation of clinical scanners for small-animal imaging with reduced resolution as compared to dedicated preclinical scanners. To overcome this disadvantage, a rat animal model was used that allows the use of higher tumour volumes. The frequently used mice tumour models only permit smaller tumour sizes for animal welfare reasons. Tumours of this size are difficult to scan with clinical scanners [5]. In contrast, the rat tumours in this study were at least two centimetres in maximal extension. The pathophysiology related to vascularisation of tumours of this size may be much more comparable to tumours in patients since diffusion distances for oxygen and other nutrients cannot cover comparatively large tumour volumes, especially at the tumour margins. Tumour sizes investigated in this study are dependent on sufficient neovascularisation – as the tumours in patients are – and should provide information more comparable to the clinical situation. In diagnostic imaging of tumours of patients, the presence of large necrotic areas (hypodense in CE-CT in the venous phase) and highly vascularised peripheral areas (hyperdense in CE-CT in the arterial phase) is a frequent clinical finding. The applicability of clinical imaging in this model is demonstrated by the comparison to the histological and immunohistological results. Furthermore, even though we pushed the imaging protocols to their limits, we believe that the results found herein are a good basis for translating this to clinical patient care.

With respect to DWI applications in oncology, the reliable transferability of ADC values between different imaging systems has been under discussion for many years [36]. The definition of standardised technical protocols for DWI and standardised interpretation of results are still under evaluation and increasing consensus is developing [37]. In our study, we used standardised parameters that are applicable for clinical imaging and allow comparability to other imaging systems. Using the comparison with histology, this research added new information to the current research on how reliable ADC values for both diffusion and perfusion are. The use of two different cell lines each with two different stages of vascular development reduced the influences on imaging due to different histological traits of different cell lines like protein expression, size, and membrane permeability of the tumour cells. Consequently, differences in imaging parameters within the same tumour cell line are more likely caused by vascularisation rather than by characteristics of the cell line. This is an important advantage compared to other research investigating histologically diverse tumour lines. The investigated lung cancer cell lines are standard in preclinical research and are previously well described. Further clinical investigations will be developed taking advantage of the results found herein.

The present study established a human tumour xenograft cancer model with modifiable vascularisation and successfully evaluated parameters reflecting vascularisation in tumours by noninvasive clinical imaging. Results were correlated with histology and immunohistology. The amount of necrosis and the pathologic tumour vasculature in tumours were visualised using clinical scanners and were correlated with histological results. The results of the study showed that DWI is capable of assessing information not only on cell density but also indirectly on the vascularisation in tumours. The differentiated observation of DWI parameters increased the information obtainable from imaging and provide additional parameters that can be introduced to large-data analysis. ADC_diff_ should be interpreted in its minimal, mean and maximal extension. The harness of established clinical methods (DWI, CE-CT, TOF) may by expanded by a better understanding of these imaging parameters. The results of this study could contribute to this. This may give more information concerning the aggressiveness of tumours, which may influence therapy [4, 32, 33]. Additionally, this study showed that clinical imaging units may well be used for preclinical imaging, which allows faster translation of results.

## Abbreviations

ADC: Apparent diffusion coefficient
ADC_diff_: Apparent diffusion coefficient with diffusion-weighted b-values
ADC_perf_: Apparent diffusion coefficient with perfusion-influenced b-values
CE-CT: Contrast-enhanced computed tomography
CM: Contrast medium
DWI: Diffusion-weighted magnetic resonance imaging
FGF: Fibroblast growth factor
MRI: Magnetic resonance imaging
MVD: Microvessel density
NSCLC: Non-small-cell lung cancer
RGE: Rat glomerular endothelial cells
rHu: Recombined human
TOF: Time-of-flight
VEGF: Vascular endothelial growth factor
